# Cardiometabolic Health Among Adult Offspring of Hypertensive Pregnancies: The Cardiovascular Risk in Young Finns Study

**DOI:** 10.1161/JAHA.117.006284

**Published:** 2018-01-06

**Authors:** Robyn J. Tapp, Alun D. Hughes, Mika Kähönen, Tien Yin Wong, Nicholas Witt, Terho Lehtimäki, Nina Hutri‐Kähönen, Pinki Sahota, Markus Juonala, Olli T. Raitakari

**Affiliations:** ^1^ Melbourne School of Population and Global Health University of Melbourne Australia; ^2^ School of Clinical and Applied Sciences Leeds Beckett University Leeds United Kingdom; ^3^ Department of Optometry and Vision Sciences The University of Melbourne Australia; ^4^ Institute of Cardiovascular Science University College London London United Kingdom; ^5^ Department of Clinical Physiology Tampere University Hospital and the University of Tampere Finland; ^6^ Singapore National Eye Center Singapore & Ophthalmology and Visual Sciences Academic Clinical Program Duke‐NUS Medical School National University of Singapore Singapore; ^7^ National Heart and Lung Institute Imperial College London London United Kingdom; ^8^ Department of Clinical Chemistry Fimlab Laboratories and School of Medicine University of Tampere Finland; ^9^ Department of Pediatrics University of Tampere and Tampere University Hospital Tampere Finland; ^10^ Research Centre of Applied and Preventive Cardiovascular Medicine University of Turku Finland; ^11^ Turku University Hospital Turku Finland; ^12^ Department of Clinical Physiology and Nuclear Medicine Turku University Hospital Turku Finland

**Keywords:** cardiac, health outcomes, microvascular dysfunction, Epidemiology, Hypertension, Blood Pressure, Imaging

## Abstract

**Background:**

Cardiometabolic health among adult offspring of hypertensive disorders of pregnancy (HDP) is relatively unknown. We hypothesized that offspring of HDP would have abnormalities in the retinal microvasculature and cardiac structure by midadulthood.

**Methods and Results:**

The Cardiovascular Risk in Young Finns Study included randomly selected children from 5 Finnish university cities. The mean age of participants was 40 years (range 34–49 years) at the time of retinal photography and cardiac assessment. Offspring born ≥37 weeks of gestation and appropriate for gestational age (n=1006) were included. Offspring of HDP had higher systolic blood pressure (β=4.68, *P*<0.001), body mass index (β=1.25, *P*=0.009), and waist circumference (β=0.25, *P*=0.042), compared with offspring of normotensive pregnancies. However, no differences in fasting glucose, insulin, lipid profile, carotid intima media thickness, or brachial artery flow‐mediated dilatation were shown. Retinal arteriolar diameters were narrower (β=−0.43, *P*=0.009) and longer (β=32.5, *P*=0.023) and the arteriolar length‐to‐diameter ratio was higher (β=2.32, *P*=0.006) among offspring of HDP, after adjustment for age and sex. Left atrial volume indexed to body surface area (β=1.34, *P*=0.040) was increased. Adjustment for the confounding effects of birth weight, body mass index, smoking and socioeconomic status, and the mediating effect of hypertension had little impact on the associations.

**Conclusions:**

Abnormalities of the retinal microvasculature and cardiac structure are seen in offspring of HDP in midadulthood. These findings may need to be considered in future primary prevention strategies of cardiovascular disease among offspring of HDP.

**Clinical Perspective**

**What Is New?**

- Cardiometabolic health among adult offspring of hypertensive disorders of pregnancy, is relatively unknown.
- This study has shown that abnormalities of the retinal microvasculature and cardiac structure are seen in offspring of hypertensive disorders of pregnancy in midadulthood.

**What Are the Clinical Implications?**

- These findings may need to be considered in future primary prevention strategies of cardiovascular disease among offspring of hypertensive disorders of pregnancy.

## Introduction

Hypertensive disorders of pregnancy ([HDP] including pre‐eclampsia and gestational hypertension) are common and associated with adverse outcomes among the offspring.^1^, ^2^ It is well established that offspring of women with HDP have higher blood pressure (BP) from childhood,^3^ with 1 recent study showing that BP trajectories remain consistently higher throughout adolescence.^4^ Evidence of early‐onset hypertension has recently been observed. In a study by Davis et al, 30% of 20‐year‐olds with hypertensive BP were born following a hypertensive pregnancy,^5^ and the lifetime risk of developing cardiovascular disease (CVD) was estimated to be 2.5 times higher, equivalent to a 40% greater risk of developing CVD.^5^

There is little evidence of structural damage from higher BP among offspring of HDP, as most studies have assessed a limited set of measures in children and young adults.^6^ In the general population, derangements in cardiac structure^7^, ^8^, ^9^, ^10^ and the retinal microvasculature^11^, ^12^ in both adults and children have been observed with elevated BP. These indicators of target organ damage including left ventricular (LV) hypertrophy, increased left atrial volume, and narrower retinal arteriolar diameters, have been associated with an increased risk of myocardial infarction and CVD mortality.^13^, ^14^, ^15^, ^16^ However, studies have not assessed these associations among offspring of HDP in midadulthood, where the risk of CVD is increased. These associations are of particular importance given the strong links with CVD events and the need for appropriate treatment at critical points in the disease process.

To address these gaps in knowledge between HDP and cardiometabolic health, we assessed the associations in the Cardiovascular Risk in Young Finns study, an adult cohort with in‐depth measures over 40 years. We hypothesized that offspring of HDP would have abnormalities in the retinal microvasculature and cardiac structure by midadulthood, and these associations would be independent of the mediating effect of current hypertension status.

## Methods

The Cardiovascular Risk in Young Finns Study is an ongoing epidemiological study of atherosclerosis risk factors from childhood to adulthood. In 1980, children and adolescents aged 3 to 18 years were invited to participate (n=4320). The study was carried out in 5 Finnish university cities and their rural surroundings, with subjects chosen randomly from the national population register from these areas.^17^ Subjects and their parents completed a detailed questionnaire, including information on birth weight and preterm birth.^18^ Birth before 37 weeks' gestation was defined as preterm birth. Term births were classified as appropriate for gestational age when birth weight was in the 50th to 90th percentile for the population. HDP was defined as hypertension only during pregnancy. The current study included 1006 participants born at term (≥37 weeks) and appropriate for gestational size, who underwent retinal photography and cardiac assessment during the 2011–2012 clinic. In the current study, our final analysis included the participants who had information on retinal parameters or cardiac parameters, BP, medication used for BP measured in 2011, and data on HDP.

Forty‐five‐degree digital retinal images centered on the macula of each eye were captured using a Canon nonmydriatic retinal camera (Canon CR6‐45NM) fitted with a Canon 10D digital single lens reflex camera attachment. One observer, blinded to subject data, graded the retinal images. A semi‐automated grading system was used to capture a range of retinal geometric parameters. Measured parameters included the (1) arteriolar and venular diameters, (2) arteriolar‐to‐venular ratio, (3) arteriolar bifurcation angles, (4) length/diameter ratios of arteriolar segments and arteriolar/venular diameter ratios (these parameters provide measures of arteriolar narrowing that are relatively unaffected by differences in optical refraction), (5) arteriolar tortuosity (estimated as the actual length of the vessel divided by the straight line distance between bifurcations minus 1), and (6) arteriolar optimality ratio and optimality deviance. Optimality ratio is the ratio of sum of “daughter” arteriolar diameters divided by the “parent” arteriolar diameter corrected for asymmetry.^19^, ^20^ For a theoretically optimal bifurcation, the optimality ratio should be 0.79, and the optimality deviance was calculated as the absolute value of the optimality ratio minus 0.79. Reproducibility of this technique is high and the average absolute difference and SD between measurements of arteriolar diameter was 0.0±0.4 pixels, consistent with previous reports.^20^ The arteriolar and venular diameters were measured at a series of intensity cross‐sections normal to the vessel at 2‐pixel intervals along the entire length of the vessel segment. At each cross‐section, the vessel diameter was measured to subpixel accuracy using a sliding linear regression filter technique as described previously and an average was calculated for each vessel.^21^

Transthoracic echocardiograms were performed according to American and European guidelines.^22^, ^23^ Sonographers from each site were trained in cardiac echocardiography according to the study protocol. Transthoracic echocardiograms were performed with Acuson Sequoia 512 (Acuson, Mountain View, CA, USA) ultrasonography, using a 3.5‐MHz scanning frequency phased‐array transducer. Analysis of the echo images were performed by a single observer using the ComPACS 10.7.8 analysis program (MediMatic Solutions, Genova, Italy). Standard echocardiographic views were obtained in all participants (parasternal long and short axis, and apical 4‐chamber). Complete 2‐dimensional, M mode echocardiographic measurements, continuous and pulsed‐wave Doppler, and mitral annulus tissue Doppler velocities were performed. Left ventricular mass and chamber area/volumes were indexed to body surface area (BSA), as per the European Society of Cardiology guidelines. The formula used to calculate BSA was DuBois and DuBois ([weight] kg to the power of 0.425×[height] m to the power of 0.725×0.007184).^24^

Ultrasound measures of carotid intima media thickness (IMT) were undertaken in 2007.^25^ The measures were performed with a Sequoia‐512, ultrasound mainframe (Acuson, Mountain View, CA, USA). All measurements were performed by the same technician, blinded to participant details. The image, from the left common carotid artery, was focused on the posterior wall. To derive mean carotid IMT, at least 4 measurements were taken ≈10 mm proximal to the carotid bifurcation. Intra‐individual reproducibility was assessed resulting in an average absolute difference and SD between measurements of 0.05±0.04 mm.^26^ Brachial ultrasound studies were performed using Sequoia 512 ultrasound mainframes (Acuson) with 13.0 MHz linear array transducer. To assess brachial artery flow‐mediated dilatation (FMD), the left brachial artery diameter was measured both at rest and during reactive hyperemia. Increased flow was induced by inflation of a pneumatic tourniquet placed around the forearm to a pressure of 250 mm Hg for 4.5 minutes, followed by a release. Three measurements of arterial diameter were performed at end‐diastole at a fixed distance from an anatomic marker at rest and 40, 60, and 80 seconds after cuff release. The vessel diameter in scans after reactive hyperemia was expressed as the percentage relative to resting scan (100%). The greatest value between 40 and 80 seconds was used to derive the maximum FMD. The 3‐month‐between‐visit coefficient of variation was 3.2% for brachial artery diameter measurement and 26.0% for FMD measurement.^25^

Height and weight were measured, and body mass index kg/m^2^ (BMI) calculated. BP measurements were obtained using a random zero sphygmomanometer. Three measurements of BP were recorded and the mean value of these was presented. Hypertension was defined as systolic BP >140 mm Hg, diastolic BP >90 mm Hg, or taking antihypertensive medication. A self‐administered questionnaire was used to determine current health status (medication and medical conditions), smoking status, and employment. Participants who indicated they were regular smokers (smoke daily) were classified as smokers. Venous blood samples were drawn after an overnight fast. Serum total cholesterol and triglycerides were determined according to the Lipid Research Clinics Program, and high‐density lipoprotein cholesterol was analyzed following precipitation of apolipoprotein‐B‐containing lipoproteins with heparin‐manganese.^27^ The homeostatic model assessment index was calculated as insulin (μU/m)×(glucose [mmol/L]/22.5).^28^

The study complied with the Declaration of Helsinki, was approved by the local ethics committees, and all subjects gave written informed consent.

### Statistical Methods

The data analysis was performed with Stata 12.0 IC (Stata Corp LP, College Station, TX, USA). Descriptive information for each variable was derived and distributions were assessed to determine normality. Data are presented as mean (SD), percentages, or median and (interquartile range). Univariate associations were assessed using ANOVA for metric variables and χ^2^ for categorical variables. The associations between HDP with CVD risk factors, retinal microvascular, and cardiac measures were analyzed using regression models. Interaction analysis between sex and offspring of hypertensive disorders of pregnancy were run for each outcome measure and none were significant; therefore data were not analyzed separately for males and females. Three models were developed. Model 1 regressed outcome variables (separately, each retinal microvascular and cardiac measure) on HDP adjusted for age and sex. Model 2 further adjusted for the potential confounding effects of birth weight, BMI, smoking, and socioeconomic status, and model 3 further adjusted for the possible mediating effect of current hypertension. Statistical significance was inferred as a 2‐sided probability at the 95% level of confidence. In order to provide a graphical illustration of the associations between vascular measures and systolic BP, quartiles were created after exclusion of those with hypertension: 1/83 to 108 mm Hg, 2/109 to 116 mm Hg, 3/117 to 126 mm Hg, and 4/>126 mm Hg and stratified by gestational status. Multivariate analysis of variance was used to assess the difference between and within groups.

## Results

The characteristics of offspring participants, stratified by HDP status, are shown in Table 1. Offspring of HDP had a higher BMI and waist circumference, systolic and diastolic BP, and a higher percentage were on medication for hypertension, compared with offspring of normotensive pregnancies. There were no significant differences in sex, smoking, socioeconomic status, high‐density lipoprotein cholesterol, total cholesterol, apolipoprotein A1, apolipoprotein B, glucose, or insulin, between the groups.

**Table 1:** General Characteristic of the Offspring According to Maternal HDP Status

	Offspring of Women Without HDP	Offspring of Women With HDP	*P* Value
N	877	129	
Age, y	41.6 (5.0)	39.7 (4.6)	<0.001
Female, %	51.2	48.8	0.185
Birth weight, g	3666 (402)	3746 (458)	0.039
BMI, kg/m^2^	26.3 (5.0)	27.3 (5.4)	0.047
BSA, m^2^	1.92 (0.22)	1.97 (0.21)	0.012
Systolic BP, mmHg	117 (14)	121 (15)	0.003
Diastolic BP, mmHg	74 (10)	77 (11.0)	0.002
BP medication, %	7.6	16.4	0.001
HDL cholesterol, mmol/L	1.34 (0.34)	1.27 (0.3)	0.065
Total cholesterol, mmol/L	5.12 (0.93)	5.14 (0.98)	0.742
Apo A1, mmol/L	1.59 (0.25)	1.55 (0.22)	0.113
Apo B, mmol/L	1.04 (0.28)	1.06 (0.03)	0.271
Glucose, mmol/L	5.4 (0.9)	5.3 (0.66)	0.935
Diabetes mellitus, %	3.2	1.6	0.306
Insulin, mU/L	9.2 (12.4)	9.8 (9.3)	0.616
Regular smoking, %	13	13	0.990

Apo indicates apolipoprotein; BMI, body mass index; BP, blood pressure; BSA, body surface area; HDL, high‐density lipoprotein; HDP, hypertensive disorders of pregnancy.

After adjustment for age and sex, offspring of HDP had higher systolic and diastolic BP, BMI, and waist circumference (Table 2). Further adjustment for confounding factors (model 2) had little impact on the regression coefficients for BMI and waist circumference and modestly reduced the regression coefficients for systolic and diastolic BP, although both remained statistically significant. After adjustment for HDP, the association with BMI and waist circumference were no longer significant.

**Table 2:** Multiple Regression of HDP Status on Offspring CVD Risk Factors

	Model 1		Model 2		Model 3	
	Regression Coefficient HDP	*P* Value	Regression Coefficient HDP	*P* Value	Regression Coefficient HDP	*P* Value
Outcome measures						
Systolic BP, mmHg	4.68 (2.27 to 7.09)^a^	<0.001^a^	3.83 (1.53 to 6.13)^a^	0.001^a^	b	
Diastolic BP, mmHg	3.48 (1.63 to 5.32)^a^	<0.001^a^	2.72 (0.98 to 4.46)^a^	0.002^a^	b	
Heart rate, bpm	0.49 (−1.30 to 2.28)	0.595	0.21 (−1.56 to 1.98)	0.817	−0.46 (−2.22 to 1.29)	0.603
BMI, kg/m^2^ b	1.25 (0.31 to 2.18)^a^	0.009^a^	1.20 (0.26 to 2.13)^a^	0.012^a^	0.59 (−0.32 to 1.50)	0.203
Waist circumference, cm	0.25 (0.01 to 0.48)^a^	0.042^a^	0.24 (0.01 to 0.47)^a^	0.049^a^	0.09 (−0.14 to 0.32)	0.456
HDL cholesterol, mmol/L	−0.04 (−0.09 to 0.02)	0.165	−0.02 (−0.07 to 0.04)	0.516	−0.01 (−0.07 to 0.04)	0.622
Total cholesterol, mmol/L	0.07 (−0.10 to 0.24)	0.440	0.05 (−0.13 to 0.21)	0.631	0.05 (−0.12 to 0.23)	0.549
Apo A1, mmol/L	−0.02 (−0.07 to 0.02)	0.269	−0.01 (−0.06 to 0.03)	0.531	−0.01 (−0.06 to 0.03)	0.519
Glucose, mmol/L	0.01 (−0.15 to 0.17)	0.857	−0.04 (−0.19 to 0.11)	0.608	−0.08 (−0.23 to 0.08)	0.316
HOMA IR	0.16 (−1.19 to 1.50)	0.821	−0.35 (−1.64 to 0.94)	0.599	−0.57 (−1.86 to 0.72)	0.387

Model 1 adjusted for age and sex, Model 2 adjusted for age, sex, birth weight, BMI, smoking, and SES. Model 3 adjusted for age, sex, birth weight, BMI, smoking, SES, and hypertension. Apo indicates apolipoprotein; BMI, body mass index; BP, blood pressure; bpm, beats per minute; CVD, cardiovascular disease; HDL, high‐density lipoprotein; HDP, hypertensive disorders of pregnancy; HOMA IR, homeostasis model assessment insulin resistance; SES, socioeconomic status.

a Where the risk factor is the primary variable of interest, it was not entered into the model as a covariate.

b Where the risk factor is the primary variable of interest, it was not entered into the model as a covariate.

Table 3 shows the effect of HDP status on offspring vascular outcomes. Retinal arteriolar diameters were narrower (β=−0.43, *P*=0.009) and longer (β=32.5, *P*=0.023), the arteriolar length‐to‐diameter ratio was higher (β=2.32, *P*=0.006), and the arteriolar‐to‐venular ratio decreased among offspring of hypertensive pregnancies, after adjustment for age and sex. Adjustment for the confounding effects of birth weight, BMI, smoking, and socioeconomic status, and the mediating effect of hypertension had little impact on the associations with retinal architecture measures. No association was observed for FMD or IMT. The analysis for FMD was re‐run using diameter after hyperemia as the outcome and baseline as the covariate. It made little difference to the outcome. The associations between FMD and offspring of hypertensive disorders of pregnancy was not significant (β=1.01 [−0.02 to 0.04] *P*=0.482), after adjustment for age and sex. Adjustment for the confounding effects of birth weight, BMI, smoking, and socioeconomic status (β=0.004 [−0.02 to 0.03] *P*=0.750), and the mediating effect of hypertension (β=0.01 [−0.02 to 0.03] *P*=0.661), had little impact on the association.

**Table 3:** Association Between HDP Status and Offspring Vascular Outcomes

	Model 1		Model 2		Model 3	
	Regression Coefficient O‐HDP	*P* Value	Regression Coefficient O‐HDP	*P* Value	Regression Coefficient O‐HDP	*P* Value
Outcome retinal microvasculature measures						
Arteriolar diameter (pixels)	−0.43 (−0.76 to −0.11)	0.009	−0.39 (−0.72 to −0.07)	0.017	−0.36 (−0.69 to 0.04)	0.030
Venular diameter (pixels)	−0.11 (−0.61 to 0.39)	0.674	−0.11 (−0.61 to 0.40)	0.675	−0.11 (−0.62 to 0.39)	0.657
Arteriolar tortuosity (10^2^)	−0.01 (−0.03 to −0.00)	0.073	−0.01 (0.03 to 0.00)	0.062	0.02 (0.03 to −0.00)	0.039
Venular tortuosity (10^3^)	−0.05 (−0.13 to 0.04)	0.279	−0.05 (−0.14 to 0.03)	0.222	−0.05 (−0.14 to −0.03)	0.199
Arteriolar length (pixels)	32.5 (4.60 to 60.45)	0.023	34.5 (6.51 to 62.53)	0.016	32.5 (4.29 to 60.74)	0.024
Venular length (pixels)	6.23 (−6.47 to 18.93)	0.336	5.64 (−7.09 to 18.38)	0.385	5.32 (−7.52 to 18.16)	0.416
Arteriolar length/diameter ratio	2.32 (0.66 to 3.98)	0.006	2.40 (0.74 to 4.05)	0.005	2.23 (0.56 to 3.90)	0.009
Venular length/diameter ratio	0.35 (−0.41 to 1.11)	0.368	0.32 (−0.44 to 1.08)	0.410	0.31 (−0.45 to 1.08)	0.422
Optimality ratio	−2.05 (−4.99 to 0.90)	0.173	−1.92 (−4.89 to 1.04)	0.204	−2.34 (−0.32 to 0.65)	0.125
Arteriolar‐to‐venular ratio	−0.03 (−0.05 to 0.001)	0.027	−0.03 (−0.05 to 0.01)	0.027	−0.02 (−0.05 to −0.01)	0.047
Outcome vascular measures						
FMD, mm^a^	−0.07 (−1.03 to 0.89)	0.885	−0.35 (−1.30 to 0.60)	0.465	−0.34 (−1.30 to 0.61)	0.480
IMT, mm^a^	0.005 (−0.012 to 0.024)	0.528	0.004 (−0.014 to 0.023)	0.650	0.003 (−0.016 to 0.021)	0.789

Model 1 adjusted for age and sex, Model 2 adjusted for age, sex, birth weight, body mass index (BMI), smoking, and socioeconomic status (SES). Model 3 adjusted for age, sex, birth weight, BMI, smoking, SES, and hypertension. FMD indicates flow‐mediated dilatation; HDP, hypertensive disorders of pregnancy; IMT, intermedia thickness; O‐HDP, offspring of hypertensive disorders of pregnancy.

a Measured in 2007.

Associations between HDP status and offspring cardiac measures are shown in Table 4. Left atrial volume indexed to BSA (β=1.34, *P*=0.040) and peak A wave (β=2.56, *P*=0.016) were higher and the ratio of early diastolic mitral annulus velocity to mitral annulus velocity associated with atrial contraction (e′/a′ ratio; β=−0.06, *P*=0.005) was lower among offspring of HDP after adjustment for age and sex (Table 4). Adjustment for the confounding effects of birth weight, BMI, smoking, and socioeconomic status (model 2) reduced the association with left atrial (LA) volume indexed to BSA, and peak A wave. Further adjustment for the mediating effect of hypertension had little impact on the regression coefficients for LA volume indexed to BSA and revealed an association with LV end‐systolic area indexed to BSA. The associations with transmitral Doppler A wave and e′/a′ ratio was no longer significant after adjustment for the mediating effect of current hypertensive status.

**Table 4:** Association Between HDP Status and Offspring Cardiac Measures

	Model 1		Model 2		Model 3	
	Regression Coefficient O‐HDP	*P* Value	Regression Coefficient O‐HDP	*P* Value	Regression Coefficient O‐HDP	*P* Value
LV structural measures						
LV diameter, diastole, cm	0.02 (−0.06 to 0.11)	0.591	−0.02 (−0.10 to 0.06)	0.568	−0.02 (−0.10 to 0.06)	0.654
LV diameter, systole, cm	−0.02 (−0.09 to 0.06)	0.701	−0.03 (−0.11 to 0.05)	0.413	−0.03 (−0.10 to 0.05)	0.506
LV ejection fraction, %	−0.18 (−0.84 to 0.47)	0.581	−0.15 (−0.81 to 0.51)	0.651	0.10 (−0.76 to 0.57)	0.777
LV mass index, g/m^2^	−0.48 (−2.91 to 1.94)	0.696	−0.83 (−3.25 to 1.59)	0.500	−0.81 (−3.24 to 1.62)	0.514
Relative wall thickness	0.01 (−0.02 to 0.40)	0.477	0.01 (−0.02 to 0.04)	0.476	0.01 (−0.02 to 0.04)	0.475
LV area end‐systole indexed BSA, mL/m^2^	0.29 (−0.02 to 0.61)	0.069	0.29 (−0.03 to 0.61)	0.078	0.33 (0.01 to 0.65)	0.044
LA volume indexed BSA, mL/m^2^	1.34 (0.06 to 2.61)	0.040	1.14 (−0.13 to 2.41)	0.079	1.29 (0.01 to 2.57)	0.049
Transmitral Doppler						
E wave, cm/s	1.66 (−0.55 to 3.87)	0.140	1.64 (−0.56 to 3.85)	0.144	1.77 (−0.46 to 3.99)	0.119
A wave, cm/s	2.56 (0.49 to 4.64)	0.016	2.08 (0.09 to 4.07)	0.041	1.55 (−0.43 to 3.54)	0.125
E/A ratio	−0.05 (−0.13 to 0.02)	0.137	0.04 (−0.11 to 0.03)	0.267	−0.02 (−0.09 to 0.05)	0.545
Tissue Doppler						
Systolic velocity (s′), cm/s	0.38 (−0.27 to 1.04)	0.246	0.44 (−0.21 to 1.10)	0.186	0.49 (−0.17 to 1.15)	0.148
Early diastolic velocity (e′), cm/s	−0.26 (−0.76 to 0.24)	0.311	−0.15 (−0.64 to 0.35)	0.555	0.03 (−0.46 to 0.52)	0.909
Late diastolic velocity (a′), cm/s	0.54 (−0.05 to 1.13)	0.074	0.44 (−0.14 to 1.03)	0.138	0.42 (−0.17 to 1.01)	0.165
Mean E/e′ ratio	0.17 (−0.01 to 0.35)	0.068	0.13 (−0.05 to 0.31)	0.158	0.08 (−0.10 to 0.26)	0.367
Mean e′/a′ ratio	−0.06 (−0.11 to −0.02)	0.005	−0.05 (−0.09 to −0.01)	0.025	−0.03 (−0.08 to 0.01)	0.110

Model 1 adjusted for age and sex. Model 2 adjusted for age, sex, birth weight, body mass index (BMI), smoking, and socioeconomic status (SES). Model 3 adjusted for age, sex, birth weight, BMI, smoking, SES, and hypertension. BSA indicates body surface area; HDP, hypertensive disorders of pregnancy; LA, left atria; LV, left ventricular; O‐HDP, offspring of hypertensive disorders of pregnancy.

The association between arteriolar diameter and offspring of HDP after adjustment for age, sex, birth weight, BMI, smoking, socioeconomic status, and the mediating effect of systolic BP was slightly weaker (β=−0.28 [−0.60 to 0.04] *P*=0.082) than when adjusted for hypertensive status. A similar pattern was evident for arteriolar length (β=30.40 [2.35–58.50] *P*=0.034), arteriolar length‐to‐diameter ratio (β=2.04 [0.38–3.69] *P*=0.016), arteriolar‐to‐venular ratio (β=−0.02 [−0.05 to 0.001] *P*=0.067), LV area end‐systole indexed to BSA (β=0.26 [−0.06 to 0.58] *P*=0.115), and LA volume indexed to BSA (β=0.99 [−0.29 to 0.2.27] *P*=0.0.128).

**Figure 1:**
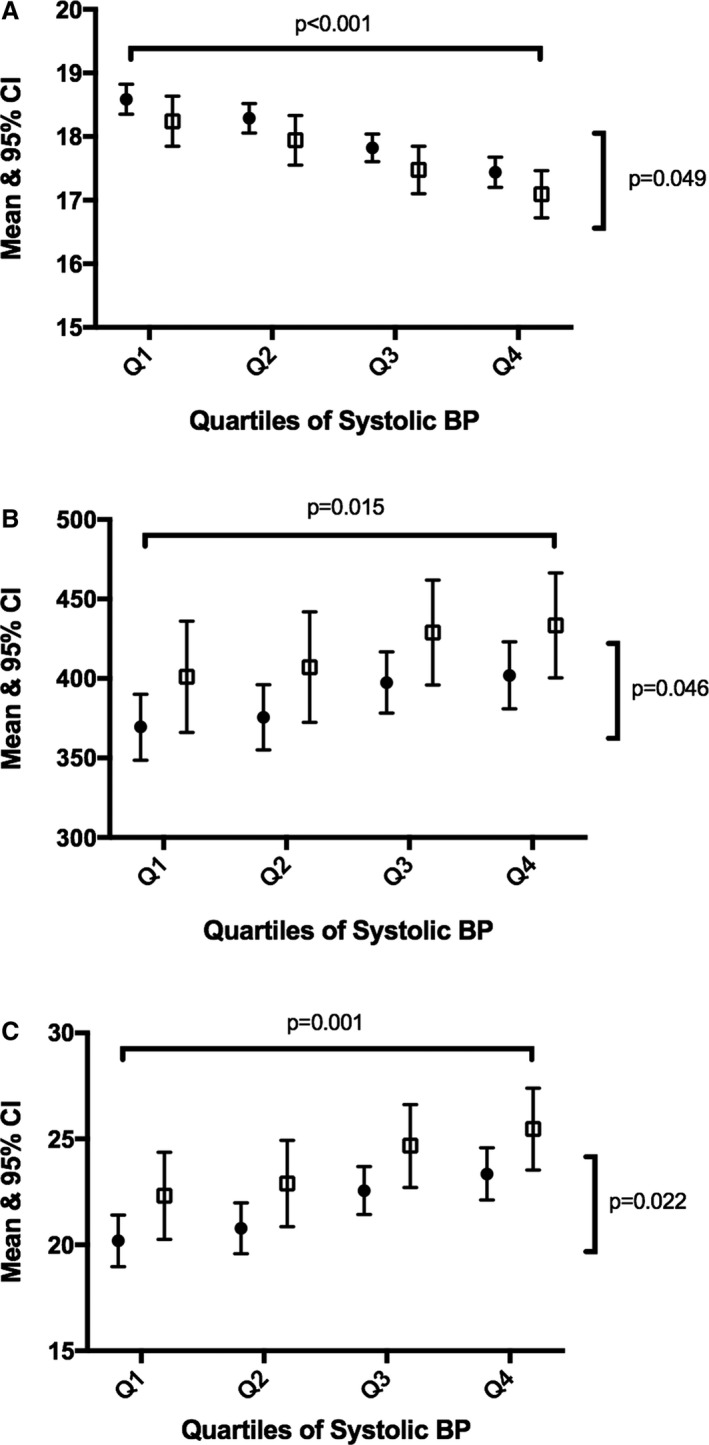
Association between quartiles of systolic BP and retinal microvascular measures by HDP status. A, Retinal arteriolar diameter. B, Retinal arteriolar length. C, Retinal arteriolar length/diameter ratio. BP indicates blood pressure; CI, confidence interval; HDP, hypertensive disorders of pregnancy.

**Figure 2:**
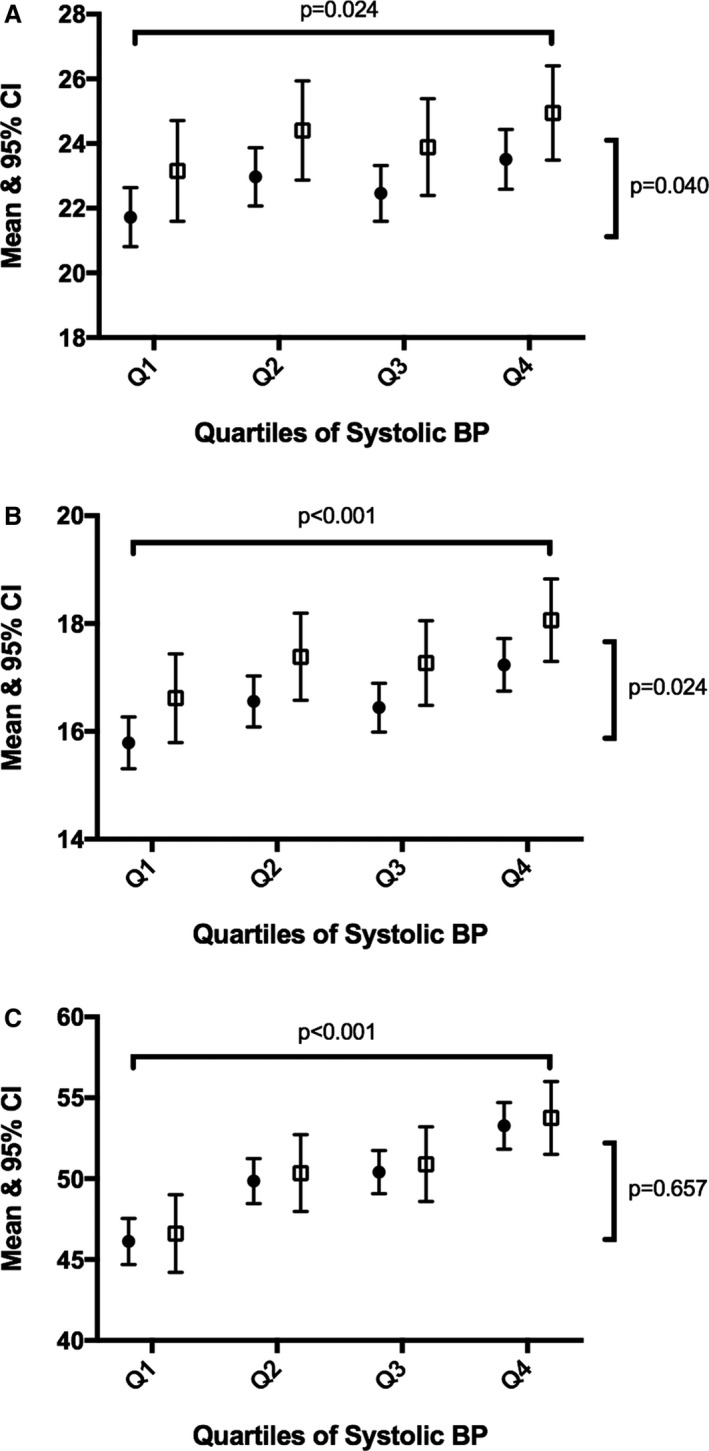
Association between quartiles of systolic BP and cardiac measures by HDP. A, Left atrium volume indexed to BSA. B, LV area end‐systole. C, Transmitral Doppler A wave. BP indicates blood pressure; BSA, body surface area; CI, confidence interval; HDP, hypertensive disorders of pregnancy; LV, left ventricular.

Figure 1 shows the age‐ and sex‐adjusted mean and 95% confidence interval for retinal microvascular measures by quartiles of systolic BP. Those on current treatment for hypertension were excluded from this analysis. For arteriolar diameter, there was a negative association with systolic BP among both groups and in each quartile of systolic BP, offspring of HDP had narrower diameters. A similar pattern of association was observed for arteriolar length and arteriolar length/diameter ratio, except the offspring of HDP had longer arterioles and a higher length/diameter ratio compared with those of a normotensive pregnancy. Figure 2 shows the age‐ and sex‐adjusted mean and 95% confidence interval for cardiac measures by quartiles of systolic BP. Both LV end‐systolic area and LA volume indexed to BSA values were higher in each quartile of systolic BP among offspring of hypertensive pregnancies. No association was observed for transmitral A wave, where the association was mediated by current systolic BP.

## Discussion

This is the first study to comprehensively examine the cardiometabolic effects of HDP among adult offspring. The study showed that offspring of women with HDP had higher BMI and waist circumference, systolic and diastolic BP, as well as evidence of adverse changes in the retinal microvasculature and cardiac structure. However, no evidence of an association with glucose, insulin, lipid profile, IMT, or FMD was found. Our study indicates that HDP are not only associated with higher BP among offspring, but also with abnormalities of the retinal microvascular and to a lesser degree cardiac structure by midadulthood, independent of risk factors and adjustment for the mediating effect of current hypertensive status.

Previous studies have assessed vascular markers in offspring of HDP (including FMD and pulse wave velocity) with mixed results; however, this is the first study to assess measures of the retinal microvasculature and cardiac structure in midadulthood using measures that are very closely associated with BP. We have shown that adult offspring of HDP have a worse retinal microvascular profile and some early alterations in their cardiac profile, compared with offspring from a normotensive pregnancy (Figures 1 and 2). Although not previously shown among offspring of HDP, the ALSPAC (Avon Longitudinal Study of Parents and Children) did speculate that associations with a wide range of vascular outcomes may emerge in adulthood.^6^ Although hypertension is the preferred measure in the current study, as on treatment BP may not capture BP load, the models were run for the measures showing a significant association after adjustment for hypertension. Although the associations were weakened, the same pattern of association was observed.

There are 2 possible mechanisms as to why HDP is associated with the microvascular and cardiac abnormalities. HDP may cause abnormalities in microvascular and the cardiac system or HDP may be a marker/consequence of abnormalities in the microvascular/cardiac system. We are unable to distinguish between these and existing evidence is limited and inconclusive.^29^ The implications of these findings independent of hypertensive status are 2‐fold. A single measure of BP may be inadequate to capture the lifetime load of BP, and target organ damage better reflects lifetime BP load, which may be higher in HDP offspring. Alternatively, using arteriolar narrowing as an example, it may be a causal mechanism of elevated BP that precedes hypertension. From a hemodynamic perspective, narrow arterioles (if widespread) would result in increased systemic vascular resistance, a characteristic feature of hypertension. There is some evidence that arteriolar narrowing is a risk factor for development of hypertension.^30^ A recent meta‐analysis showed that each 20‐μm decrease in retinal caliber was associated with 1.12 mm Hg increase in systolic BP over 5 years. The study findings were consistent with the hypothesis that generalized microvascular dysfunction precedes the onset of hypertension.^31^ In addition, LA volume is strongly associated with elevated BP and obesity. It is also an indicator of increased filling pressure over time, in a similar way that hemoglobin A_1c_ is a measure of blood glucose over time. It is also a powerful predictor of CVD outcomes.^16^ Although change in LA volume indexed to BSA was observed in the present study, other evidence of abnormalities of diastolic function was limited and further evaluation at a later time point is warranted, particularly given a study in mice has shown that gestational hypertension can lead to LV hypertrophy in offspring, with further injections of isoproterenol (used to induce cardiac stress) resulting in LV diastolic dysfunction (increased E/A ratio and E/e′) and interstitial myocardial fibrosis.^32^

ALSPAC assessed offspring FMD, pulse wave velocity, and brachial artery diameter (age 9 to 12 years) and showed no association with gestational hypertension. Similarly, vascular outcomes in subgroups born preterm were the same as offspring born at term.^6^ Consistent with the findings of ALSPAC, this study assessed FMD at age 40 years and found no association between HDP and FMD despite higher BP in offspring of HDP. FMD has previously been observed to predict incident hypertension over an ≈8‐year follow‐up period in older individuals (average age 60 years) in the Framingham Heart Study Offspring cohort.^33^ Whether these differences are attributable to differences in ages between studies or some other factor is unknown.

Other studies, from more selected, smaller samples have shown evidence of an association with pulmonary pressure, carotid IMT, and FMD. A study by Lazdam et al assessed a sample of 71 participants born preterm and showed that offspring of a hypertensive pregnancy had increased carotid IMT and 30% lower FMD at age 24 years.^34^ The present study excluded those born preterm and small for gestational age from the main analysis, as it has been demonstrated that being born small for gestational age and in particular preterm birth are associated with changes in the retinal microvascular,^35^ cardiac structure, IMT, and FMD.^18^ So although impaired fetal growth and preterm birth contribute to the mechanisms of early vascular changes, they are not the primary mechanistic link responsible for the observed abnormalities among offspring of HDP in the present study. In a small study of 138 women living at high altitude, pulmonary pressure was 30% higher and FMD 30% lower in offspring of mothers with preeclampsia. The study went further to assess whether dysfunction was related to preeclampsia or to a genetic abnormality that predisposes the mother to preeclampsia and the offspring to vascular alterations. They assessed the vascular function of offspring of mothers with preeclampsia; the offspring were born following a normotensive pregnancy. The study showed no impairment in FMD or pulmonary artery pressure in this group, suggesting that preeclampsia might induce epigenetic alterations leading to vascular dysfunction in the offspring. Epigenetic modifications are thought to play a central role in the programming of adult disease.^36^ Other possible mechanisms include shared genetics, which may better explain the isolated association with maternal and offspring elevated BP shown in the larger cohort studies,^3^ or direct intrauterine effects related to placental dysfunction in association with HDP.

The present study has several limitations. We were only able to assess HDP and not the subcategories of long‐term hypertension in pregnancy, preeclampsia/eclampsia, and gestational hypertension separately, which would provide a greater understanding of the differences between the conditions. Limited information on the mother's prepregnancy characteristic was available. These may have provided additional information on the maternal risk factor profile and their contributions to adverse outcomes. This was an observational study, with loss to follow‐up over many years,^37^ and while loss to follow‐up may compromise the representativeness of the sample, it is unlikely that it would account for the associations observed.

## Conclusions

Abnormalities of the retinal microvasculature and cardiac structure are seen in offspring of HDP. These findings may need to be considered in future primary prevention strategies of cardiovascular disease among offspring of HDP.

## Sources of Funding

The Young Finns Study has been financially supported by the Academy of Finland: grants 134309 (Eye), 126925, 121584, 124282, 129378 (Salve), 117797 (Gendi), 41071 (Skidi), and 286284, the Social Insurance Institution of Finland, Kuopio, Tampere and Turku University Hospital Medical Funds, Juho Vainio Foundation, Paavo Nurmi Foundation, Finnish Foundation of Cardiovascular Research and Finnish Cultural Foundation, Tampere Tuberculosis Foundation, and Emil Aaltonen Foundation. Hughes received support from a Biomedical Research Centre Award to University College London Hospital and from the British Heart Foundation (CS/15/6/31468, PG/12/29/29497).

## Disclosures

None.
